# One-Year Clinical Outcomes of Water Vapor Thermal Therapy for Benign Prostatic Hyperplasia: A Single-Center Experience of 52 Patients

**DOI:** 10.7759/cureus.95347

**Published:** 2025-10-24

**Authors:** Takashi Okabe

**Affiliations:** 1 Urology, Mizuhodai Urology, Medical Corporation Koshin-Kai, Saitama, JPN

**Keywords:** benign prostatic hyperplasia, day surgery, rezum, urology, water vapor thermal therapy

## Abstract

Introduction

Water vapor thermal therapy (WVTT) has emerged as a minimally invasive surgical therapy (MIST) for benign prostatic hyperplasia (BPH) management. While international studies have confirmed its safety and efficacy, real-world data from Japanese outpatient clinics remain scarce. This study aimed to evaluate the one-year clinical outcomes of WVTT performed in a single urology clinic, representing the first Japanese single-center report describing one-year clinical outcomes of outpatient WVTT.

Methods

We conducted a retrospective cohort study at Mizuhodai Urology, including 52 patients who underwent WVTT between March 2023 and September 2024. Baseline and follow-up assessments at one, three, six, and 12 months included quality of life (QOL, International Prostate Symptom Score (IPSS)-QOL), prostate volume (PV), and postvoid residual (PVR) volume. Adverse events (AEs) were classified using the Clavien-Dindo system. Risk factors for the restart of BPH medication and failure of the first trial of void (TOV) were analyzed using multivariate logistic regression.

Results

Significant improvements in QOL, PV, and PVR were maintained throughout the 12-month follow-up period. All procedures were completed as day-case interventions under spinal anesthesia, with a median operative time of 3 min and a mean hospitalization duration of 259 min. Catheter removal was achieved in all patients, although the first TOV failed in seven cases. Restart of BPH medication was required in seven patients after a median of 339 days. Multivariate analysis identified PV ≥60 mL as a risk factor for medication restart and PV ≥70 mL for first TOV failure. AEs occurred in six patients within 14 days, all grade I-II, with no grade ≥III events.

Conclusion

Clinic-based WVTT was safe and effective for BPH management, with sustained improvement in QOL, PV, and PVR at one year. To the best of our knowledge, this is the first Japanese study reporting one-year outcomes of WVTT. These results support its feasibility as a minimally invasive, office-based therapy while underscoring the importance of patient selection in larger prostates.

## Introduction

Benign prostatic hyperplasia (BPH) is a highly common cause of lower urinary tract symptoms (LUTS) in aging men, significantly impacting quality of life [1,2]. Approximately 40% of men over 50 years of age experience BPH-related symptoms [3]. Although pharmacotherapy with α-blockers, 5-alpha reductase inhibitors, and other agents benefits many patients, a substantial proportion ultimately require surgery. Transurethral resection of the prostate (TURP) remains the gold standard [4] but is associated with perioperative risks, including bleeding, erectile or ejaculatory dysfunction, incontinence, and potential long-term complications [5].

The demand for minimally invasive, outpatient procedures has increased due to both medical and socioeconomic factors, including reduced healthcare costs and greater patient convenience [6]. Alternatives, such as holmium laser enucleation of the prostate (HoLEP) and photoselective vaporization of the prostate (PVP), are well-established [7,8] while minimally invasive surgical therapies (MISTs), including prostatic urethral lift (UroLift), water vapor thermal therapy (WVTT), and prostatic artery embolization (PAE), have gained increasing attention for symptom relief with fewer complications and a shorter recovery period [9,10]. Among these MISTs, WVTT using the Rezūm platform delivers transurethral injections of 103°C convective steam into the prostate and has shown proven safety and efficacy in LUTS secondary to BPH [11].

An earlier Japanese study of 40 patients treated with WVTT in an office-based outpatient clinic demonstrated feasibility, safety, and short-term efficacy, with improvements in prostate volume (PV), postvoid residual (PVR) volume, and International Prostate Symptom Score-Quality of Life (IPSS-QOL) score at three months [12]. However, a longer follow-up period and evaluation of prognostic factors are needed to validate its clinical role.

This study aimed to evaluate the one-year clinical outcomes of WVTT for BPH performed in a single outpatient urology clinic in Japan. Specifically, the study assessed changes in functional outcomes (IPSS-QOL, PV, and PVR volume) at one, three, six, and 12 months after treatment, as well as the incidence of adverse events (AEs) and predictive factors for re-medication and trial of void (TOV) failure using multivariate logistic regression analysis.

## Materials and methods

Study design and setting

This retrospective cohort study was conducted at Mizuhodai Urology, a single-unit urology clinic in Fujimi City, Saitama, Japan. The Rezūm system (Boston Scientific, Marlborough, MA, USA) was introduced at the clinic in March 2023. All procedures were performed as day-case surgeries under spinal anesthesia, following previously described techniques [11,13]. 

Patient selection

A total of 52 patients who underwent WVTT between March 2023 and September 2024 were included. All patients were male and met the inclusion criteria for symptomatic BPH (IPSS-QOL ≥4 and PV 30-100 cm³), representing typical candidates for WVTT. Patients with active or recent urinary tract infection (UTI), prior invasive prostate intervention, or suspected prostate cancer (based on elevated prostate-specific antigen (PSA) or Prostate Imaging Reporting and Data System (PI-RADS) ≥3 on MRI) were excluded to avoid confounding influences on postoperative outcomes. All 52 patients were included in the final analysis, and no patients were lost to follow-up during the 12-month observation period. A summary of the inclusion and exclusion criteria is provided in Table 1.

**Table 1:** Inclusion and exclusion criteria BPH, benign prostate hyperplasia; IIPSS-QOL, International Prostate Symptom Score quality of life; UTI, urinary tract infection; PSA, prostate-specific antigen; PI-RADS, Prostate Imaging Reporting and Data System; PV, prostate volume

Inclusion criteria	
1	Male subjects >50 years of age who have symptomatic BPH
2	IPSS-QOL score ≥4
3	PV >30 cm^3^ to ≤100 cm^3^
Exclusion criteria	
1	Active or history of UTI within the past 3 months
2	Any prior invasive prostate intervention
3	Suspicious of prostate cancer due to elevated PSA or PI-RADS ≥3 on MRI

Procedure details

The Rezūm procedure was performed using the Rezūm system, which delivers 103°C water vapor into hyperplastic prostate tissue via transurethral injection. The procedure followed the standard technique previously described by McVary et al. [11] and Okabe T [12]. The number of injections (range four to seven) was determined according to the prostate size and the presence of a median lobe, with each injection lasting approximately 9 seconds. Vapor was applied from the bladder neck to the verumontanum along both lateral lobes, with additional injections to the median lobe when present. All procedures were completed as outpatient day-case surgeries under spinal anesthesia. 

Follow-up schedule and data collection

As in our previous report [12], patient data, including operative time, postoperative observation time, IPSS-QOL score, PV, PVR volume, catheterization status, medication use, and AEs, were assessed at baseline and at one, three, six, and 12 months after treatment. The IPSS-QOL was evaluated according to the standardized IPSS system [14]. PVR was measured by transabdominal ultrasonography following established guidelines [15]. AEs occurring within 14 days were classified according to the Clavien-Dindo system [16]. The first TOV was performed according to institutional protocol [12]: approximately one week (±2 days) post-procedure in non-retention cases, and four weeks (±2 days) in patients with preoperative retention. TOV success was defined as a PVR <300 mL measured by ultrasound.

Statistical analysis

Continuous variables were summarized using descriptive statistics. Normally distributed data were expressed as mean ± SD, and non-normally distributed data as median with interquartile range (IQR). Temporal changes from baseline to follow-up were analyzed using repeated-measures ANOVA with Bonferroni post hoc correction for pairwise comparisons. Potential predictors of restart of BPH medication and first TOV failure were analyzed using multivariate logistic regression, including clinically relevant covariates such as PV, number of injections, and preoperative urinary retention status. A two-tailed p<0.05 was considered statistically significant. All analyses were performed using JASP software (version 0.18.3; JASP Team; https://jasp-stats.org/).

## Results

Patient characteristics

Fifty-two patients were included in this study. The median age was 72.0 years (IQR, 51.0-84.0). The mean PV was 50.6±14.7 mL. Preoperative catheterization, history of urinary retention, and presence of a median lobe were observed in 17.3%, 11.5%, and 32.7% of the patients, respectively (Table 2).

**Table 2:** Patient characteristics IQR, interquartile range; BPH, benign prostatic hyperplasia; IPSS, International Prostate Symptom Score; QOL, quality of life; PVR, postvoid residual volume; PV, prostate volume

Patient characteristic	Patients (N=52)
Age in years, median (IQR)	72.0 (51.0-84.0)
Performance status, median (IQR)	0 (0-2)
Preoperative medication for BPH, N (%)	
Alpha blocker	50 (96.1)
5-alpha reductase inhibitor	31 (59.6)
Phosphodiesterase-5 inhibitor	5 (9.6)
Anticoagulants/platelet aggregation inhibitors, N (%)	3 (5.8)
Preoperative IPSS-QOL score, mean ± SD	4.7±0.6
Preoperative PVR in mL, mean ± SD	171.9±321.0
Preoperative PV, mean ± SD	50.6±14.7
Preoperative catheterization, N (%)	9 (17.3)
History of urinary retention, N (%)	6 (11.5)
Presence of median lobe, N (%)	17 (32.7)

Perioperative data

The median operative duration was 3.0 min (IQR, 2.0-11.0). Patients received a median of five injections (IQR, 4-7), and the mean hospitalization time was 258.5±47.0 min (Table 3).

**Table 3:** Perioperative and postoperative efficacy outcomes IQR, interquartile range; TOV, trial of void; BPH, benign prostatic hyperplasia

Perioperative and postoperative efficacy outcomes	Patients (N=52)
Duration of operation in minutes, median (IQR)	3.0 (2.0-11.0)
Number of injections, median (IQR)	5.0 (4.0-7.0)
Postoperative observation time in minutes, mean ± SD	258.5±47.0
Total number of successful catheter removals, N (%)	52 (100)
Total days until successful catheter removal, median (IQR)	8.0 (6.0-87.0)
Total number of first TOV failures, N (%)	7 (13.5)
Total days until successful catheter removal in 43 patients without a catheter, median (IQR)	7.0 (6.0-22.0)
Total days until successful catheter removal in nine patients with a catheter, median (IQR)	29.0 (28.0-87.0)
Total number of discontinuations of BPH medication, N (%)	51 (98.0)
Total days until discontinuation of BPH medication, median (IQR)	62.0 (24.0-158.0)
Total number of restarts of discontinued BPH medication, N (%)	7 (13.7)
Total days until restarting of BPH medication, median (IQR)	339.0 (70.0-797.0)

Catheter management and medication use

Catheters were successfully removed in all 52 patients (100%). The median time to removal was eight days (IQR, 6.0-87.0). In 43 patients without preoperative catheters, removal was achieved after a median of seven (IQR, 6.0-22.0). In the remaining nine patients with preoperative catheters, removal was achieved after a median of 29 days (IQR, 28.0-87.0) (Table 3).

First TOV failed in seven patients. BPH medications were discontinued after treatment by 51 of 52 patients (98.0%), with a median discontinuation time of 62 days (IQR, 24.0-158.0). However, seven of these patients required re-initiation of medication after a median time of 339 days (IQR, 70.0-797.0) (Table 3).

Risk factor analysis

Multivariate logistic regression identified PV ≥60 mL as a predictor of BPH medication restart (Table 4) and PV ≥70 mL as a predictor of first TOV failure (Table 5).

**Table 4:** Potential risk factors for restarting discontinued BPH medication OR, odds ratio; PV, prostate volume; BPH, benign prostatic hyperplasia

Potential risk factors for restarting BPH medication			
Parameters	OR	CI	p-value
PV ≥60 mL	25.657	2.174-302.792	0.010
History of urinary retention	6.016	0.283-127.789	0.250
Age ≥75	2.958	0.352-24.836	0.318
Duration of operation ≥6 min	6.086	0.141-262.904	0.374
Presence of median lobe	0.509	0.070-3.680	0.504

**Table 5:** Potential risk factors for first TOV failure OR, odds ratio; PV, prostate volume; TOV, trial of void

Potential risk factors for first TOV failure			
Parameters	OR	CI	p-value
PV ≥70 mL	14.670	1.678-128.271	0.015
History of urinary retention	1.907	0.128-28.513	0.640
Age ≥75	2.256	0.282-18.028	0.443
Duration of operation ≥6 min	5.166	0.202-131.826	0.320
Presence of median lobe	3.052	0.277-33.594	0.362

Functional outcomes

**Figure 1:**
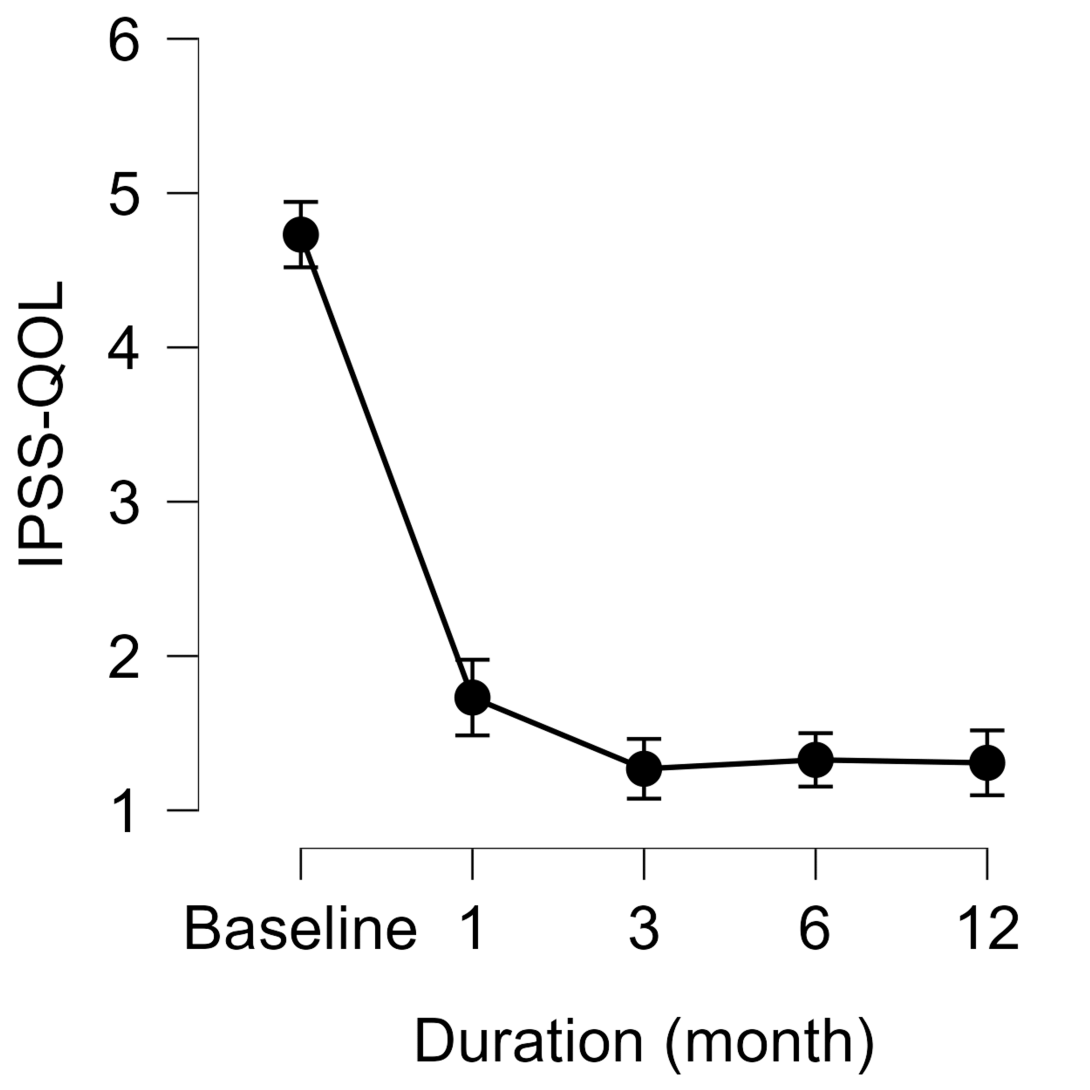
Changes in IPSS-QOL from baseline to 12 months after WVTT IPSS-QOL at baseline and at one, three, six, and 12 months after WVTT. Values are presented as means with 95% CI. Significant improvements were observed at all time points compared with baseline. IPSS-QOL, International Prostate Symptom Score-quality of life; WVTT, water vapor thermal therapy

**Figure 2:**
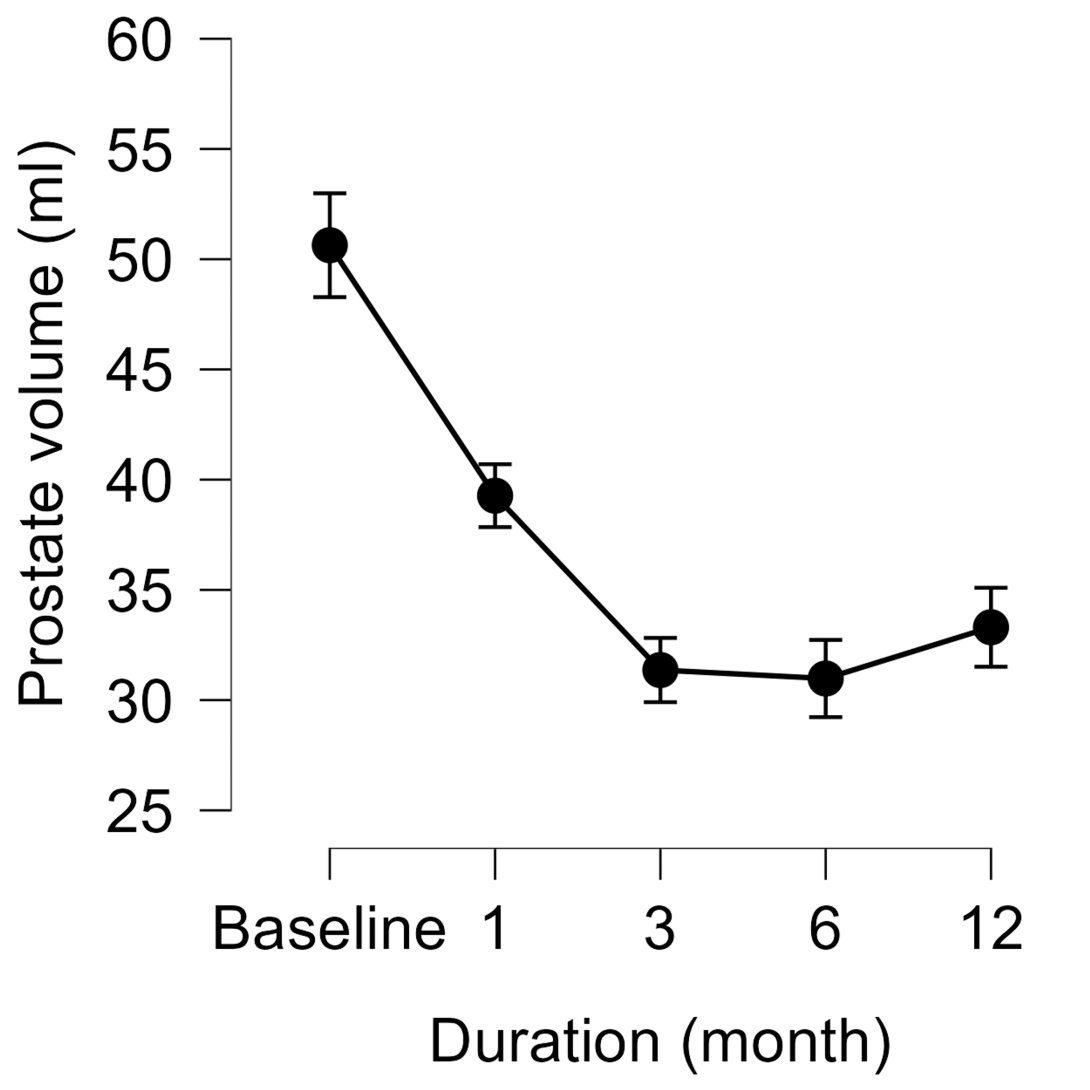
Changes in PV from baseline to 12 months after WVTT PV at baseline and at one, three, six, and 12 months after WVTT. Values are presented as means with 95% CI. A significant reduction from baseline was sustained throughout follow-up. PV, prostate volume; WVTT, water vapor thermal therapy

**Figure 3:**
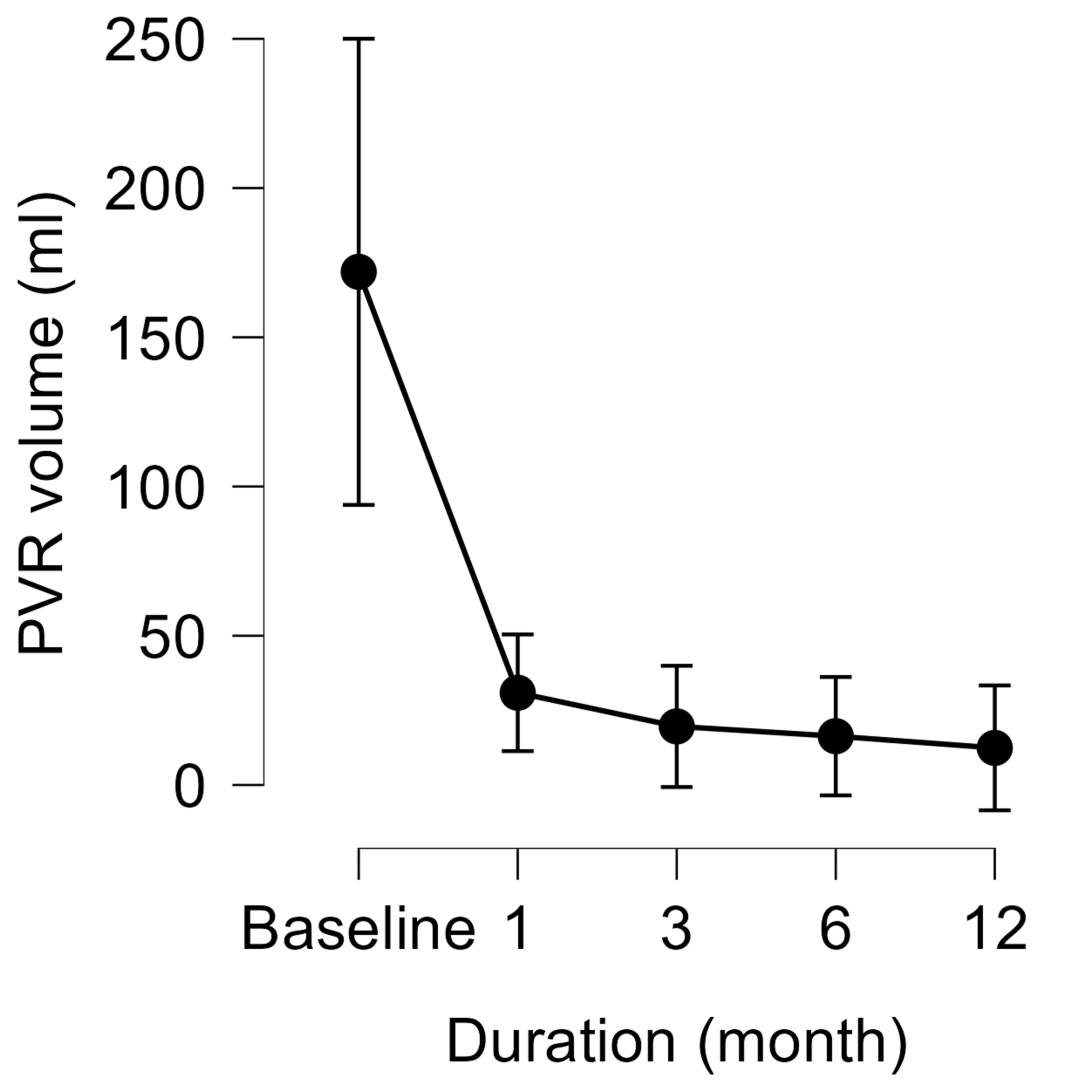
Changes in PVR volume from baseline to 12 months after WVTT PVR volume at baseline and at 1, 3, 6, and 12 months after WVTT. The Y-axis minimum was adjusted to 0 mL to reflect the true physiological range. Values are presented as means with 95% CI. PVR decreased significantly compared with baseline and remained stable thereafter. PVR, postvoid residual; WVTT, water vapor thermal therapy

WVTT yielded significant functional improvements. QOL, assessed using IPSS-QOL, decreased markedly from baseline and remained significantly improved at all follow-up time points. PV decreased significantly at one, three, six, and 12 months, with the largest reduction observed at three months. PVR decreased sharply at one month and remained low thereafter. Repeated-measures ANOVA confirmed significant overall changes in all three parameters (p<0.001), with Bonferroni post hoc tests showing significant differences between baseline and all follow-up time points (Figures 1-3).

Safety outcomes

AEs occurred in six patients within 14 days after WVTT. These included urinary tract pain in three patients (grade II in one (1.9%) and grade I in two (3.8%)) and UTI in the remaining three patients (5.8%, all grade I). No Clavien-Dindo grade ≥III events were reported (Table 6).

**Table 6:** Postoperative AEs observed within 14 days AEs, adverse events; UTI, urinary tract infection

	Patients (N=52)	
	Clavien-Dindo classification	
AEs, N (%)	Ⅰ	Ⅱ
UTI	3 (5.8)	0
Urinary tract pain	2 (3.8)	1 (1.9)

## Discussion

This retrospective study demonstrated that WVTT is safe and effective in an outpatient clinic setting. All 52 patients underwent the procedure as a day-case intervention under spinal anesthesia, with a median operative time of three minutes and a mean hospitalization of approximately four hours. Catheter removal was achieved in all patients, and AEs were limited to mild urinary symptoms or low-grade infections. Importantly, no Clavien-Dindo grade ≥III complications occurred, confirming the feasibility and safety of WVTT in a real-world, office-based practice.

The outpatient applicability of WVTT aligns with international reports highlighting its role as a MIST suitable for office or day surgery practice. Elterman et al. described WVTT and UroLift as office-based procedures that reduce hospitalization and healthcare costs while maintaining safety and efficacy [9]. Kaltsas et al. further emphasized Rezūm’s favorable safety profile, with most AEs being mild and transient [17]. Similarly, Wolters et al. demonstrated consistent improvements in IPSS, PVR, and PV in a real-world cohort [18]. Our findings are consistent with these observations, supporting the integration of WVTT into Japanese outpatient clinical practice.

Compared with traditional inpatient surgeries, such as TURP, HoLEP, and PVP, all of which require longer operative times and hospitalization [19,20], WVTT provides a less invasive alternative that can be performed in an outpatient setting, with lower risks of bleeding and sexual dysfunction [9]. The absence of major complications and successful catheter removal in all patients in this study highlights its value among MISTs.

Prostrate volume was identified as an important prognostic factor, with larger glands (≥60-70 mL) associated with medication restart and first TOV failure. This suggests that prostate size should be considered carefully during patient selection and perioperative counseling for WVTT.

Previous reports support this observation. Elterman et al. noted that larger prostates, though treatable with WVTT, may yield less durable responses [9]. Long-term follow-up studies have also indicated that prostate size influences the persistence of symptoms and retreatment rate [21]. In contrast, other MISTs, such as UroLift, are generally recommended for prostates <80 mL, reflecting technical limitations in large glands [22]. Reviews of office-based procedures further emphasize that efficacy may diminish in very large prostates, for which more invasive options, particularly HoLEP, remain superior [23].

Altogether, these findings highlight the need for a tailored, volume-based strategy in selecting candidates for MISTs. WVTT is safe and effective for appropriately chosen patients, but those with very large prostates should be counseled regarding slower recovery and possible additional therapy.

This study has several limitations. First, it was conducted at a single center with a relatively small sample size, which may limit the generalizability of the findings. Second, the absence of a control group prevents direct comparison with other treatment modalities such as HoLEP or PVP. Nevertheless, this real-world, clinic-based analysis provides valuable prospective data on one-year outcomes following WVTT in a Japanese outpatient setting, demonstrating consistent functional improvements and a favorable safety profile.

Taken together, WVTT demonstrated significant and sustained improvement in both objective and subjective outcomes at one year in a real-world outpatient setting, with a favorable safety profile. However, due to the retrospective, single-center nature of the study and the absence of a comparator group, these findings should be interpreted within the context of the study’s limitations. Further multicenter prospective studies with larger cohorts and comparative analyses are warranted to validate the efficacy and generalizability of these results.

## Conclusions

WVTT was safe and effective in improving LUTS due to BPH in a single-center Japanese outpatient cohort, with sustained improvements in PV, PVR, and QOL at one year. PV was identified as a key predictor of prognosis, underscoring the importance of careful patient selection. WVTT represents a minimally invasive treatment that complements existing surgical modalities, particularly for patients seeking outpatient, low-risk interventions.
